# Editorial Department

**Published:** 1856-02

**Authors:** 


Ex-Prof. M. L. Knapp, of Cincinnati, is an industrious man. With every full moon we receive a pamphlet from him, embodying some new discovery in medicine. He would be a miracle of industry, were it not that he is not necessitated to leave his arm-chair to obtain his facts. We are wrong. He was informed by a hotel keeper in Pittsburg that vegetables were very expensive there during the cholera of 1854, ergo, cholera is a manifestation of scorbutus, depending upon a short supply of succulent vegetables! This discovery constituted the subject matter of his first pamphlet. Since then he has put several new saddles on the old hobby, and announced an equal number of similar discoveries. Stomatitis materna is a manifestation of scurvy; cholera infantum is a manifestation of scurvy; and if he goes on at this rate we shall have the whole nosology reduced to scurvy; as homeopathy has already reduced it to itch.

